# Brain magnetic resonance imaging findings in Mitochondrial Neurogastrointestinal Encephalomyopathy (MNGIE): A case-based review

**DOI:** 10.1016/j.radcr.2024.11.047

**Published:** 2024-12-12

**Authors:** Maria Veatriki Christodoulou, Nikoletta Anagnostou, Anastasia K. Zikou

**Affiliations:** Department of Radiology, Medical School, University of Ioannina, Stavros Niarchos Avenue, Ioannina 45500, Greece

**Keywords:** Mitochondrial neurogastrointestinal encephalomyopathy, MNGIE, TYMP mutation, Brain MRI, Leukoencephalopathy

## Abstract

Mitochondrial neurogastrointestinal encephalopathy (MNGIE) is a rare autosomal recessive disorder, manifesting with gastrointestinal dysmotility, cachexia, ptosis and peripheral neuropathy. Diffuse leukoencephalopathy in brain MRI is a hallmark of MNGIE. We report a case of a 21-year-old female with MNGIE, presenting with cachexia and chronic diarrhea. Brain MRI revealed lesions in the cerebral deep white matter and the pons, with sparing of the subcortical U-fibers and the cerebral cortex and no apparent involvement of the cerebellum, basal ganglia, and thalamus. A literature review led to the identification of 72 additional cases with MNGIE that underwent brain MRI. Leukoencephalopathy of the cerebral white matter was present in all but 2 patients. The objective of this study is to increase radiologists' awareness of this challenging-to-diagnose disease, as well as to demonstrate the value of advanced MRI techniques in understanding the underlying pathology. The presence of leukoencephalopathy on brain MRI in patients with cachexia and neurological manifestations, should raise the suspicion for MNGIE and trigger further biochemical and genetic testing.

## Introduction

### Definition and genetics

Mitochondrial Neurogastrointestinal Encephalomyopathy (MNGIE) is a very rare autosomal recessive disorder of nucleotide metabolism, caused by mutations of the TYMP gene, which codes for the thymidine phosphorylase (TP) enzyme, expressed in most human tissues. It is related to various deletions and partial depletion of mitochondrial DNA (mtDNA) resulting to deterioration of mitochondrial functions in affected cells [[1], [2], [3], [4], [5]]. MNGIE is a multisystemic disorder, affecting mainly the gastrointestinal and neurological systems [3]. In MNGIE TP deficiency results in an accumulation of thymidine and deoxyuridine substrates, consequent instability of mtDNA and mitochondrial respiratory chain damage [6,7]. TYMP mutations encountered in MNGIE patients can be homozygous or compound heterozygous and are typically loss of function mutations [1,8].

### Clinical manifestations

MNGIE has a prevalence of approximately 1-9/1000000, occuring more often in the 2nd-3rd decade of life. The disease onset may be early or late with gradual progression and rapid deterioration, with death occurring at an average age of 35-37 years [[9], [10], [11], [12], [13]]. MNGIE is a multisystemic mitochondriopathy affecting tissues with high energy needs [[14], [15], [16]]. The broad clinical spectrum is summarized in Table 1. The primary clinical manifestations of MNGIE include severe gastrointestinal dysmotility, cachexia, progressive external ophthalmoparesis with or without ptosis, peripheral neuropathy, and leukoencephalopathy [17,18]. GI dysmotility is often the initial and most prominent symptom, resulting from neuromuscular dysfunction and affecting any part of the gastrointestinal tract, resulting in gastrointestinal and respiratory complications [15,[19], [20], [21], [22], [23], [24]]. Although patients present with diffuse leukoencephalopathy, dementia or cognitive impairment are not reported [9,17]. In atypical cases, the symptoms can be misleading, with a wide range of differential diagnosis, resulting to misdiagnosis or delay of the diagnosis even up to a decade [9]. The objective of this review is to examine the significance of MRI in diagnosing MNGIE in clinical practice, particularly in instances where diagnostic challenges arise.

**Table 1:** MNGIE summary of clinical manifestations depending on the system affected.

Systems	Manifestations
**Gastrointestinal**	Dysmotility, diarrhea, cramps, borborygmi, nausea, vomiting, gastroparesis, dysphagia, constipation, cachexia, intestinal pseudo-obstruction, hepatic steatosis, cirrhosis [10,11,15,17,20,25]
**Neurological**	Diffuse leukoencephalopathy, Peripheral neuropathy (stocking-glove pattern of sensory loss, absent tendon reflexes, distal limb weakness), ptosis, ophthalmoparesis, hearing loss, dysarthria, pes cavus [11,15,17,20,25]
**Ophthalmological**	Pigmentary retinopathy [15,17]
**Cardiological**	Cardiomyopathy (rarely) [15]

### Imaging findings

Diffuse leukoencephalopathy is the hallmark of MNGIE presenting in all patients [9,11]. MRI usually shows bilateral, almost symmetrical, T2-weighted and FLAIR hyperintensity and T1-hypointensity in the white matter of the semioval centers, sparing of the U-fibers, and less frequently in the basal ganglia, the thalamus, the white matter of the cerebellum and the splenium of the corpus callosum [9,16,25,26]. The temporal lobe demonstrates a lesser incidence of affection compared to the frontal, parietal, and occipital lobes [16]. Similar findings may apply to the pons and the midbrain with variable involvement [9,11,15,23]. Despite the presence of leukoencephalopathy, no significant correlation exists between disease severity and clinical presentation with white matter lesions [11]. Cerebellar atrophy has been reported in some patients [9,19,26].

Noteworthy radiological phenomena include “T2 washout,” where areas of increased signal intensity in T2 and FLAIR sequences correspond to nearly isointense regions on Diffusion Weighted Imaging (DWI) [11]. Diffusion Tensor Imaging (DTI) reveals elevated Mean Diffusivity (MD), Radial diffusivity (RD), and Axonal Diffusivity (AD) values in parieto-occipital white matter, particularly accentuated RD values, alongside significantly decreased Fractional Anisotropy (FA) values compared to controls, a trend also observed in the optic radiation [16].

Magnetic Resonance Spectroscopy (MR-Spectroscopy) emerges as a valuable tool in mitochondrial disease evaluation, offering insights beyond conventional sequences [9]. Advancing age correlates with decreased N-Acetylaspartic acid (NAA) levels within white matter, indicative of cortical volume loss, even in the absence of cerebral atrophy [27]. MR-Spectroscopy further reveals diminished concentrations of Choline (Cho), Creatine (Cr), and NAA in affected white matter regions, suggesting neuronal, axonal, and glial cell loss [16,27]. Since there is a reduction of the three main metabolites, the ratios Cho/Cr and NAA/Cr ratios do not show a difference between healthy individuals and patients, while the absence of elevated lactate levels implies intact anaerobic glycolysis, characteristic of the disease's gradual progression [27].

### Pathophysiology

The near-complete or total loss of TYMP activity precipitates a detrimental buildup of nucleosides within tissues, disrupting the mitochondrial respiratory chain [9,10,18,28]. Structural and metabolic alterations in the white matter can be attributed to several factors, including disruption of the blood-brain barrier, microangiopathy, and energy deficit in subependymal cells, leading to elevated white matter water content [16,29]. This pronounced increase in intramyelin and intracellular water content may underpin the observed reductions in metabolite concentrations and the presence of selective or prominent RD [16]. Furthermore, white matter T2 hyperintensity is predominantly attributed to myelin abnormality rather than demyelination [11,17]. It is noted that neuropathological investigations revealed the replacement of smooth muscle fibers in vascular walls by dense fibrous tissue [16].

## Case presentation

### Clinical history

A 21-year-old woman was admitted to the gastroenterology department in May 2022 due to cachexia (BMI 10.5 kg/m^2^) and chronic diarrhea syndrome. The patient is a member of a family of 3 children, among which the older sister passed away at the age of 17, with a positive genetic testing for MNGIE. Our patient had also undergone previous genetic testing for mutations in the thymidine phosphorylase gene (TYMP-exon 6), which was positive. The subject was a T/T homozygote at the first base of exon 6 (chr22:50965712, not described to date in the international literature), which entails the change GCC=>GTC, Ala=>Val (A=>V) (Ala: nonpolar, neutral => Val: nonpolar, neutral) at codon 216 of the TYMP protein.

As part of the diagnostic procedure, a test for celiac disease and an endoscopic examination was performed and an MRI of the brain was conducted. Furthermore, a sample was sent to a Netherlands laboratory for measurement of deoxyuridine and thymidine, which showed marked elevation of the levels of both nucleosides [Thymidine 28 μmol/l (Normal Range (NR) 0-0.2), deoxyuridine 81.3 μmol/l (NR 0-0.5)].

Due to the patient's emaciation, a Hickmann catheter was placed and the patient received parenteral nutrition, while continuing conventional per os diet with her body weight gradually increasing (BMI: 12.5 kg/m^2^).

### Imaging findings

**Fig. 1:**
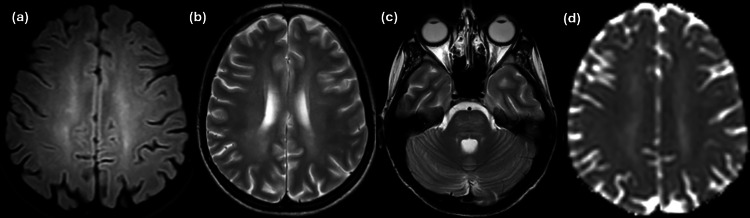
**(A).** Axial FLAIR image showing diffuse high signal intensity involving white matter of the centrum semiovale. U-Fibers are spared. (B, C). Axial T2 weighted image showing diffuse, almost symmetric high signal intensity involving periventricular and deep white matter **(B)** and pons **(C). (D)**. Apparent Diffusion Coefficient (ADC) maps reveal the involved periventricular and deep white matter areas with increased diffusivity.

**Fig. 2:**
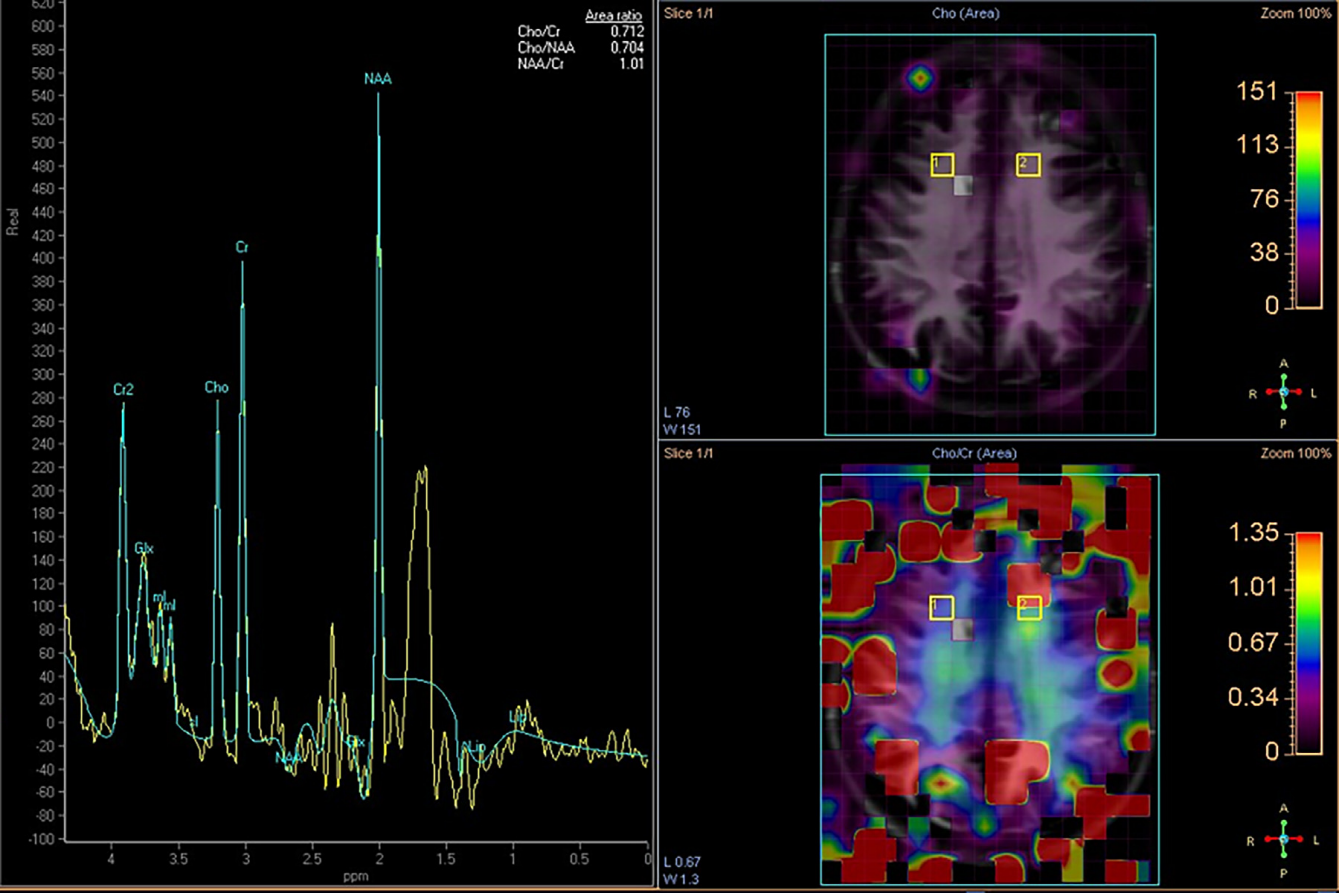
MR spectroscopy volume of interest in the frontal white matter. Metabolite ratios NAA/Cr and Cho/Cr do not differ from healthy subjects. Note the high level of noise in the patient.

Brain MRI revealed on axial T2 weighted and FLAIR -images diffusely increased signal intensity of cerebral deep and periventricular white matter (WM). The WM lesions spare subcortical U- fibers and cerebral cortex (Fig. 1A, B). The pons was also involved (Fig. 1C). Ventricular system and subarachnoid space range appeared to be increased in all conventional and WM-only sequences, probably in the context of white matter loss. Prominent Virchow-Robin spaces are seen to the centrum semiovale and brain stem. Note that in the pCASL technique, which highlights the microcirculation blood flow, blood flow was symmetrically reduced for the patient's age. There was no restriction of diffusion to the lesions (Fig. 1D). MR spectroscopy showed no pathological ratios of common metabolites (Cho, NAA, Cr, Lac, Lip) (Fig. 2).

## Methods

A systematic literature search was performed in December 2023 to identify eligible case reports and case series on brain MRI findings in mitochondrial neurogastrointestinal encephalomyopathy. We searched PubMed major database, using the following keywords: “mitochondrial neurogastrointestinal encephalomyopathy” or “MNGIE” and “brain MRI”. We did not put time limitations on publication dates, and the search was restricted by language (German, Danish, Spanish). All articles, including titles, abstracts, and full texts, were independently reviewed by two authors (NA, MVC). Information about pathophysiology and clinical aspects of MNGIE was included for a better understanding of the topic. Reference lists from eligible articles were also scrutinized to ensure the comprehensiveness of the bibliography.

**Fig. 3:**
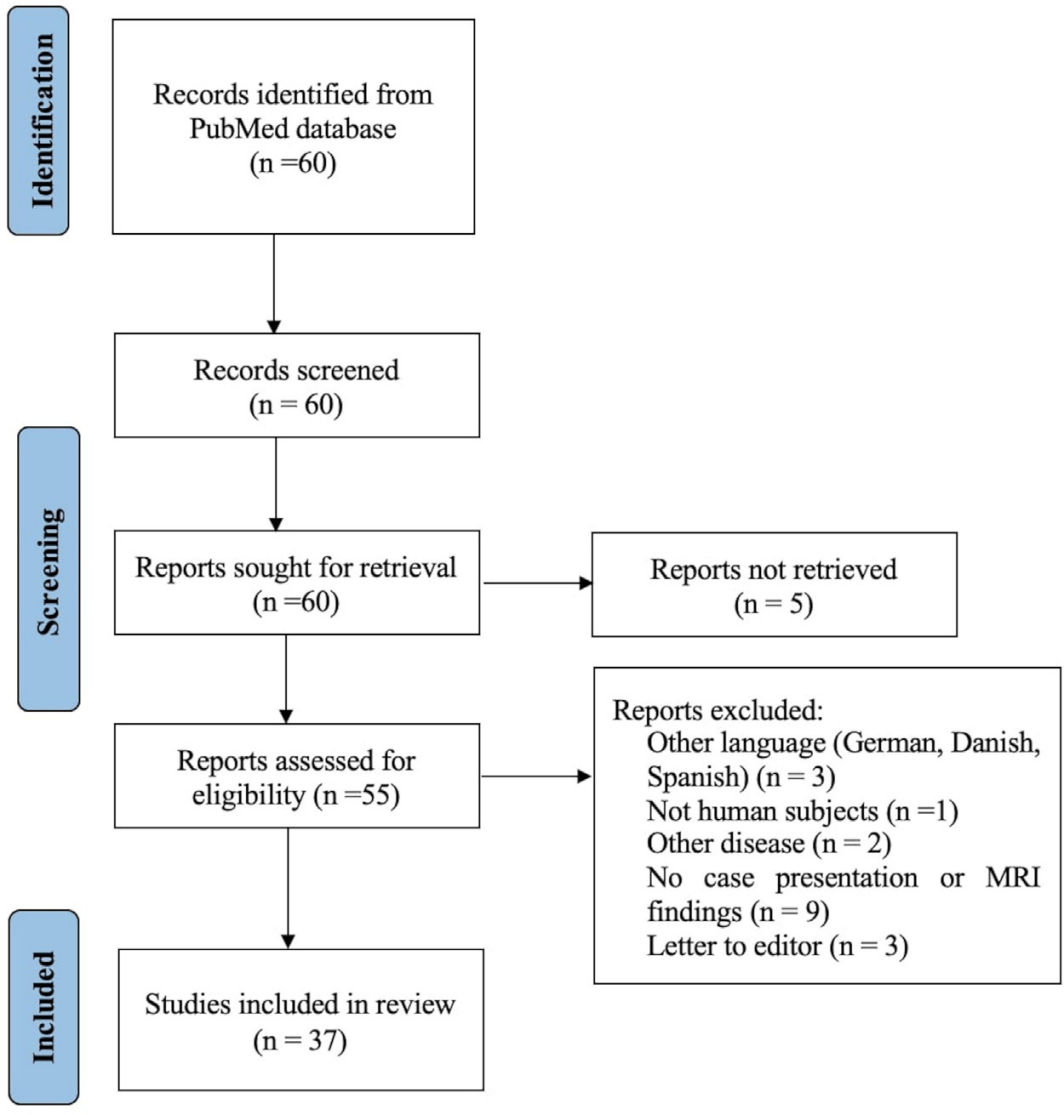
Methodology flowchart.

In the literature search, 60 publications were found. Out of those, 37 were assessed as eligible for the current study. The excluded articles referred to other diseases or MNGIE cases without available MRI findings. The method of this systematic search is shown in a flow diagram (Fig. 3). For data analysis, IBMSPSS was used.

## Results

In the literature search, 37 reports with MNGIE have been identified including a total of 72 patients [[9], [10], [11],^13^,[16], [17], [18],^20^,^22^,^23^,^26^,^27^,[30], [31], [32], [33], [34], [35], [36], [37], [38], [39], [40], [41], [42], [43], [44], [45], [46], [47], [48], [49], [50], [51], [52], [53], [54]]. The imaging findings of these patients are summarized in the Supplementary Material. Variables such as age during MRI acquisition, gender, symptoms and gene mutations have also been recorded in the Supplementary Material and discussed compared to the present case.

According to the reviewed studies (Table 2), no gender predisposition for MNGIE was observed, with patients presenting at a mean age of 26.53 (SD ± 10.3) years at the time of their initial MRI. The vast majority of patients showed leukoencephalopathy of the cerebral white matter except for only 2 patients (2.8%) [31,48]. Although specific lesion distributions were not consistently reported, involvement of the cerebellar white matter (n = 22), brainstem (n = 20), and to a lesser extent, the internal capsules (n = 14), basal ganglia-thalami (n = 15), and corpus callosum (n = 9) was commonly observed. Although U-fibers involvement is typically absent in MNGIE, a total six patients were reported [11,41]. In our patient, MRI revealed lesions in the cerebral white matter and pons, with spared U-fibers, while no involvement of the cerebellum, basal ganglia, or thalamus was observed. Brain atrophy, consistent with findings in five patients from reviewed studies, was also reported [31,47].

**Table 2:** MNGIE frequency table of lesions depending on location and presence of brain atrophy.

Location of lesions	Yes n (%)	No n (%)	Not reported n (%)
**Cerebral WM**	70 (97,2%)	2 (2,8%)	-
**Cerebellar WM**	22 (30,6%)	12 (16,7%)	38 (52,8%)
**Capsules**	14 (19,4%)	12(16,7%)	46 (63,9%)
**Corpus callosum**	9 (12,5%)	13(18,1%)	50(69,4%)
**Basal ganglia, thalami**	15 (20,8%)	14(19,4%)	43(59,7%)
**Brain stem**	20 (27,8%)	15 (20,8%)	37 (51,4%)
**U-fibers**	6(8,3%)	21 (29,2%)	45 (62,5%)
**Brain atrophy**	5 (6,9%)	7 (9,7%)	60 (83,3 %)

## Discussion

Diffusely increased signal intensities on FLAIR and T2-weighted MRI images within cerebral and cerebellar white matter are not exclusive to MNGIE but are observed across various disorders [11,26]. Toxic encephalopathies, caused by exposure to a great variety of exogenous chemical compounds, may present as diffuse symmetric white matter abnormalities. Likewise, endogenous toxins originating from inborn errors of metabolism, such as lysosomal storage diseases, may yield analogous brain lesions [55]. For instance, Krabbe disease and metachromatic leukodystrophy exhibit abnormally increased T2-weighted signal intensity in periventricular white matter, the corpus callosum, internal capsule posterior limbs, cerebellar white matter, and brainstem, sparing peripheral subcortical white matter. Conversely, Canavan's disease, another genetic leukodystrophy, showcases similar MRI findings but with early involvement of subcortical white matter and the pallidi, with relative sparing of the putamina [11].

Congenital muscular dystrophies manifest diffuse symmetric cerebral white matter hypomyelination alongside structural malformations like cortical dysplasia, cerebellar hypoplasia, cerebellar cysts, and ventriculomegaly [56]. Cerebral autosomal dominant arteriopathy with subcortical infarcts and leukoencephalopathy (CADASIL) and sporadic subcortical arteriosclerotic encephalopathy also present with diffuse symmetric leukoencephalopathy [57]. Moreover, radiation therapy can induce long-term diffuse symmetric cerebral white matter lesions, while sparing the cortex and subcortical white matter, with relative sparing of basal ganglia, internal capsule, and posterior fossa structures [11]. Brain MRI is also critical in differentiating cases of pseudomitochondrial neurogastrointestinal encephalomyopathy, which present with typical clinical manifestations, but lack signs of leukoencephalopathy or TP dysfunction [58].

MNGIE represents a devastating disease with a progressive course, often fatal if left untreated [9,59]. Current therapeutic options primarily focus on supportive care to improve nutritional status through enteral or parenteral administration [20]. Several treatment modalities have been proposed, including Hematopoietic Stem Cell Transplantation (HSCT), carrier erythrocyte-entrapped thymidine phosphorylase therapy (CEETP), liver transplantation, and hemodialysis, aimed at temporarily restoring TYMP activity and reducing toxic metabolite levels (thymidine, deoxyuridine) [20,52].

Utilization of MRI pre- and post-treatment could be crucial in assessing the reversibility of white matter lesions. In this article, we present a female patient with MNGIE alongside a comprehensive literature review. Leukoencephalopathy occurs in all patients, even with atypical clinical phenotype, and is a “cornerstone” in the differential diagnosis from other MNGIE-like disorders, underscoring the importance of MRI for accurate diagnosis and early treatment initiation, ultimately leading to improved life expectancy.

In addition to conventional MRI, advanced imaging techniques such as Arterial Spin Labeling (ASL), MR spectroscopy (MRS), and Diffusion Tensor Imaging (DTI) in MNGIE offer valuable insights into disease mechanisms and progression. ASL has shown potential in mitochondrial diseases, like MELAS syndrome, by detecting focal hyperperfusion during preclinical or acute stroke-like episodes, often before structural MRI changes appear [60,61]. Both hyperperfusion and hypoperfusion have been described in affected regions[62], suggesting variable blood flow patterns associated with mitochondrial dysfunction. In MNGIE, ASL may similarly help identify regional perfusion abnormalities, aiding in the early detection of affected brain areas.

MRS studies in MNGIE have noted biochemical changes in white matter indicative of neuronal and glial dysfunction[9,16,27] or a normal metabolic profile[17]. Scarpelli et al.[9] observed a progressive reduction in NAA and an increase in Cho on follow-up MRS, which paralleled the spread of T2 signal hyperintensities, suggesting that serial MRS imaging might serve as a biomarker for disease progression.

DTI complements conventional imaging by assessing white matter integrity at a microstructural level. Findings like T2 washout on DWI in MNGIE could indicate vasogenic interstitial edema, which aligns with the pathological findings of glial and vasogenic contributions to leukoencephalopathy[9,29].

In conclusion, given the evolving nature of MNGIE, the above imaging techniques can be valuable both initially and during follow-up to monitor disease progression and treatment response. Regular imaging could help clarify the pathophysiology of leukoencephalopathy, assess its potential for reversibility, and provide insights into therapy efficacy and the likelihood of recovery.

## Patient consent

Complete written informed consent was obtained from the patient for the publication of this study and accompanying images.

## Appendix Supplementary materials

Image, application 1
