# Supplementary material for: Brain magnetic resonance imaging findings in Mitochondrial Neurogastrointestinal Encephalomyopathy (MNGIE): A case-based review
Source: Radiol Case Rep. 2024 Dec 12;20(3):1298–305. doi: 10.1016/j.radcr.2024.11.047 (PMC11700260; doi:10.1016/j.radcr.2024.11.047)
Supplement: Supplementary file 1 [file mmc1.pdf]

Supplemental Table 1. Affected brain areas on MRI of patients with MNGIE in reviewed studies

Study	Cerebral WM	Cerebellar WM	Capsules	Corpus callosum	Basal ganglia-thalami	Brain Stem	U-fibers	DWI	ADC coefficient	Contrast enhancement	Brain atrophy
Bariş et al, 2010	Yes	Yes	N/R	N/R	Yes	Yes	N/R	N/A	N/A	N/A	N/R
Barragán et al, 2005: 6 patients	Yes: 5/6	N/R	N/R	N/R	Yes: 2/6	N/R	No	N/A	N/A	N/A	Yes: 4/6
Cardaioli et al, 2011	Yes	N/R	N/R	N/R	N/R	N/R	N/R	N/A	N/A	N/A	N/R
Carod-Artal et al, 2007	Yes	Yes	N/R	No	No	Yes	N/R	N/A	N/A	N/A	N/R
Celebi et al, 2006	Yes	Yes	N/R	N/R	Yes	Yes	N/R	N/A	N/A	N/A	N/R
Çoban et al, 2013	Yes	Yes	N/R	No	No	No	No	N/R	Increased	No	N/R
	Yes	Yes	No	No	No	No	No	N/R	Increased	No	N/R
	Yes	Yes	N/R	No	No	No	No	No restriction	Increased	No	N/R
Demaria et al, 2016	Yes	N/R	Yes	Yes	N/R	Yes	No	N/A	N/A	N/A	N/R

Durrani et al, 2019	Yes	N/R	No	No	N/R	N/R	N/R	N/A	N/A	N/A	N/R
Erdogan et al, 2019	Yes	Yes	No	No	Yes	N/R	N/R	N/A	N/A	N/A	N/R
Feddersen et al, 2009	Yes	N/R	Yes	No	Yes	Yes	N/R	N/A	N/A	N/A	N/R
Gamez & Minoves, 2006	Yes	Yes	N/R	N/R	N/R	Yes	N/R	N/A	N/A	N/A	N/R
	Yes	Yes	N/R	N/R	N/R	Yes	N/R	N/A	N/A	N/A	N/R
	Yes	N/R	N/R	Yes	N/R	N/R	N/R	N/A	N/A	N/A	N/R
	Yes	N/R	N/R	N/R	N/R	Yes	N/R	N/A	N/A	N/A	N/R
Gramegna et al, 2018	Yes	No	No	Yes	No	No	N/R	N/A	N/A	N/A	N/R
	Yes	Yes	Yes	Yes	Yes	Yes	N/R	N/A	N/A	N/A	N/R
	Yes	No	Yes	No	Yes	No	N/R	N/A	N/A	N/A	N/R
	Yes	No	No	No	No	No	N/R	N/A	N/A	N/A	N/R
	Yes	Yes	Yes	Yes	Yes	Yes	N/R	N/A	N/A	N/A	N/R
	Yes	Yes	Yes	Yes	Yes	Yes	N/R	N/A	N/A	N/A	N/R
	Yes	Yes	Yes	Yes	Yes	Yes	N/R	N/A	N/A	N/A	N/R
Khan et al, 2022	Yes	N/R	N/R	N/R	N/R	N/R	N/R	N/A	N/A	N/A	N/R
Kripps et al, 2020	Yes	N/R	N/R	N/R	N/R	N/R	N/R	N/A	N/A	N/A	N/R

	Yes	N/R	N/R	N/R	N/R	N/R	N/R	N/A	N/A	N/A	N/R
	Yes	N/R	N/R	N/R	N/R	N/R	N/R	N/A	N/A	N/A	N/R
Kučerová et al, 2018	Yes	N/R	N/R	N/R	N/R	N/R	N/R	Restricted	N/A	N/A	N/R
Li et al, 2011	Yes	Yes	N/R	N/R	Yes	Yes	N/R	N/A	N/A	N/A	N/R
Libernini et al, 2012	Yes	N/R	N/R	N/R	N/R	N/R	N/R	N/A	N/A	N/A	N/R
	Yes	N/R	N/R	N/R	N/R	N/R	N/R	N/A	N/A	N/A	N/R
Martín et al, 2004	Yes	N/R	N/R	N/R	N/R	Yes	N/R	N/A	N/A	N/A	N/R
Massa et al, 2009	Yes	N/R	N/R	N/R	N/R	Yes	Yes	N/A	N/A	N/A	N/R
Millar et al, 2004: 5 patients	Yes: 5/5	Yes: 4/5	Yes: 5/5	Yes: splenium 4/5, genu 3/5	Yes: caudate, thalami 3/5, putamen 2/5	Yes: midbrain 2/5, pons 2/5	Yes: 5/5	N/A	N/A	available in 3 patients: No enhancem ent	N/R
Nagata et al, 2017	Yes	Yes	N/R	N/R	Yes	N/R	N/R	N/A	N/A	N/A	No
Nalini et al, 2011	Yes	N/R	N/R	N/R	N/R	N/R	N/R	N/A	N/A	N/A	N/R
Oztas et al, 2010	Yes	N/R	N/R	N/R	N/R	N/R	N/R	N/A	N/A	N/A	N/R

Patel et al, 2019	Yes	N/R	N/R	N/R	N/R	N/R	No	No restriction	N/A	N/R	N/R
	Yes	N/R	N/R	N/R	N/R	N/R	No	No restriction	N/A	N/A	N/R
Peker et al, 2005	Yes	N/R	N/R	N/R	N/R	Yes	N/R	N/A	N/A	N/A	N/R
Petcharunp. et al, 2010	Yes	No	N/R	N/R	No	No	N/R	N/A	N/A	Yes	Yes
Rousset et al, 2008	Yes	No	Yes	Yes	No	No	No	N/R	Increased	No	No
Said et al, 2005	Yes	N/R	N/R	N/R	N/R	N/R	N/R	N/A	N/A	N/A	N/R
	Yes	N/R	N/R	N/R	N/R	N/R	N/R	N/A	N/A	N/A	N/R
	Yes	N/R	N/R	N/R	N/R	N/R	N/R	N/A	N/A	N/A	N/R
	No	No	No	No	No	No	No	N/A	N/A	N/A	N/R
Scarpelli et al, 2012	Yes	N/R	N/R	No	No	N/R	N/R	N/A	N/A	N/A	N/R
Scarpelli et al, 2013	Yes	No	No	N/R	N/R	Yes	No	N/A	N/A	No	N/R
	Yes	No	No	N/R	N/R	No	No	No restriction	Increased	No	N/R

	Yes	No	No	N/R	N/R	No	No	No restriction	Increased	No	N/R
	Yes	No	No	N/R	N/R	No	No	No restriction	Increased	No	N/R
Schüpbach et al, 2007	Yes	No	Yes	Yes	Yes	Yes	No	No restriction	N/A	No	No
	Yes	N/R	No	No	N/R	No	No	No restriction	N/A	No	No
	Yes	N/R	No	No	N/R	No	No	No restriction	N/A	No	No
Shaibani et al, 2009	Yes	N/R	N/R	N/R	Yes	N/R	N/R	N/A	N/A	N/A	N/R
Suh et al, 2013	Yes	Yes	N/R	N/R	Yes	Yes	N/R	N/A	N/A	N/A	N/R
Tawk et al, 2020	Yes	No	N/R	N/R	N/R	No	N/R	N/A	N/A	N/A	N/R
Wang et al, 2016	Yes	N/R	N/R	N/R	N/R	N/R	N/R	N/A	N/A	N/A	N/R
Yavuz et al, 2007	Yes	N/R	N/R	N/R	N/R	N/R	N/R	N/A	N/A	N/A	N/R
Zimmer et al, 2009	Yes	N/R	N/R	N/R	N/R	N/R	N/R	N/A	N/A	N/A	N/R

Abbreviations. N/A: Not Available, N/R: Not Reported

Supplemental Table 2. Age at MRI acquisition, Gender and Symptoms of MNGIE patients in reviewed studies

Study	Age (y) / Gender	Gastrointestinal (GI) Symptoms	Neuromuscular Symptoms
Bariş et al, 2010	18/F	abdominal pain, vomiting, diarrhea, cachexia	paresthesiae, ptosis, ophthalmoplegia, muscle atrophy
Barragán-Campos et al, 2005: 6 patients	20.0-21.8 /N/R	N/A	N/A
Cardaioli et al, 2011	21/F	abdominal pain, vomiting and diarrhoea	mild limb weakness, mild palpebral ptosis
Carod-Artal et al, 2007	35/M	epigastric pain, dysmotility, pseudoobstruction	muscle cramps, memory problems, ptosis
Celebi et al, 2006	18/F	abdominal pain, vomiting, cachexia, borborygmi	paresthesia
Çoban et al, 2013	19/F	abdominal cramping, diarrhea, vomiting, cachexia	muscle weakness, bilateral ptosis, ophthalmoplegia
	22/M	vomiting, abdominal cramping, cachexia	hearing loss, muscle weakness, bilateral ptosis
	25/M	nausea, vomiting, abdominal cramping, diarrhea	hearing loss, muscle weakness, bilateral ptosis
Demaria et al, 2016	18/F	constipation, reduced oral intake, cachexia	bilateral ptosis, diffuse muscle wasting
Durrani et al, 2019	17/M	vomiting, diarrhea, cachexia	hearing loss, vision impairment, ophthalmoparesis
Erdogan et al, 2019	19/M	abdominal pain, vomiting, pseudoobstruction	external ophthalmoplegia, peripupillar pigmentation
Feddersen et al, 2009	21/F	abdominal pain, cachexia, growth retardation	bilateral ptosis, infranuclear ophthalmoparesis
Gamez & Minoves, 2006	29/M	chronic intestinal pseudoobstruction, cachexia	external ophthalmoparesis, sensorineural hearing loss

	25/F	abdominal pain, vomiting, diarrhea, cachexia	ptosis, ophthalmoparesis, retinopathy, hearing loss
	34/F	weakness	ptosis, ophthalmoparesis
	27/F	abdominal pain, cachexia	neurological symptoms
Gramegna et al, 2018: 7 patients	23–38/ 3F, 4 M	7/7: Yes, not specified	6/7: ptosis, chronic progressive external ophthalmoplegia 1/7: peripheral neuropathy
Khan et al, 2022	23/F	epigastric pain, bilious emesis, weight loss	bilateral lower limb weakness
Kripps et al, 2020	14mo/M	vomiting	decreased muscle tone
	36/F	no GI symptoms	external ophthalmoplegia, ptosis, fatigue, memory problems
	21/F	gastroesophageal reflux, chronic abdominal pain	weakness, extremity paresthesias
Kučerová et al, 2018	26/F	diarrhea, abdominal pain, early satiety, weight loss	N/A
Li et al, 2011	28/F	"functional" gastrointestinal disorders, cachexia	bilateral hearing deterioration, unsteady gait, ptosis
Libernini et al, 2012	16/M	hyporexia, weight loss, recurring vomiting	reactive mood disorder, severe muscular atrophy
	13/M	failure to thrive	sensorineural hearing deficit, severe muscular atrophy
Martín et al, 2004	60/F	no GI symptoms (only subclinical gastroparesis)	bilateral ptosis, tremor, bilateral external

			ophthalmoplegia
Massa et al, 2009	67/F	melana, early satiety, vomiting	diffuse muscle atrophy, ataxia, ophthalmoplegia, ptosis
Millar et al, 2004:<br>5 patients	34–46/<br>4M, 1F	4/5: severe gastrointestinal dysmotility, diarrhea<br>1/5: abdominal pain	5/5: ptosis, ophthalmoparesis, peripheral neuropathy
Nagata et al, 2017	20/F	weight loss, abdominal pain	N/A
Nalini et al, 2011	32/M	recurrent vomiting, early satiety, weight loss	ptosis, restricted eye movements, muscle wasting
Oztas et al, 2010	21/M	weight loss, cachexia	ophthalmoplegia, ptosis, ataxia, sensory neuropathy
Patel et al, 2019	25/F	diarrhea, vomiting, abdominal pain	N/A
	34/F	diarrhea, lethargy, borborygmi	bilateral ptosis, mild waddling gait
Peker et al, 2005	25/M	frequent gastroenteritis, growth retardation	trigeminal neuralgia, bilateral ptosis
Petcharunpaisan et al, 2010	26/M	abdominal pain, nausea, irregular bowel habits	weakness, bilateral ptosis, diffuse muscle weakness
Rousset et al, 2008	14/F	vomiting, epigastric pain, weight loss, cachexia	no opthalmological abnormalities or neuropathy
Said et al, 2005	25/F	abdominal pain, vomiting, diarrhea	bilateral ptosis, gait impairment, sensory-motor deficit
	25/M	gastroparesis, diarrhea, abdominal pain	upward gaze limitation, bilateral pes cavus
	24/F	diarrhea and occlusions, progressive cachexia	weakness of lower limb, lateral gaze limitation, pes cavus
	27/F	chronic intestinal obstruction, diarrhea	paresthesiae of the hands and feet, walking difficulty,

			ataxia
Scarpelli et al, 2012	24/M	gastrointestinal dysmotility, severe cachexia	mild generalized weakness, diffuse muscle atrophy
Scarpelli et al, 2013	33/F	enteropathic arthritis	partial bilateral blepharoptosis, diffuse muscle atrophy
	38/M	alternating bowel function	eyelid ptosis, muscle cramps
	26/M	atypical GI symptoms	severe neuropathy
	22/F	abdominal pain, diarrhea, vomiting, intestinal pseudo-obstruction, cachexia	bilateral lid ptosis and ophthalmoplegia, sensorineural deafness, diffuse muscle atrophy
Schüpbach et al, 2007	22/M	pseudo-obstruction, abdominal pain, diarrhea	bilateral ptosis, severe muscular atrophy
	20/M	occasional borborygmi	slight muscle atrophy, fluctuating ptosis on the left
	20/M	diarrhea, vomiting, flatulence, cachexia	severe muscular atrophy
Shaibani et al, 2009	42/F	gastrointestinal dysmotility, cachexia	ophthalmoplegia, ptosis, peripheral neuropathy, dysarthria
Suh et al, 2013	28/F	abdominal pain, diarrhea, fever, headaches	ptosis, external ophthalmoparesis, pigmentary retinopathy
Tawk et al, 2020	40/M	diarrhea, abdominal pain, vomiting, weight loss	bilateral ptosis, ophthalmoplegia, sensorineural hearing loss

Wang et al, 2016	48/M	weight loss, elevated stool frequency, dysphagia	visual loss, external ophthalmoplegia and ptosis
Yavuz et al, 2007	15/F	vomiting, epigastric pain, cachexia	muscle weakness, atrophy, bilateral ophthalmoplegia, ptosis
Zimmer et al, 2009	35/F	diarrhea, cachexia	left-sided ptosis, external ophthalmoplegia, mild sensory loss

Abbreviations. F: female, M: male, y: years, mo: months, GI: gastrointestinal

Supplemental Table 3. Gene mutations of MNGIE patients in reviewed studies

Study	Genes
Bariş et al, 2010	novel homozygous TYMP gene mutation c.112G>T
Barragán-Campos et al, 2005: 6 patients	N/A
Cardaioli et al, 2011	homozygous c.1249dupC TYMP mutation
Carod-Artal et al, 2007	homozygous mutation C4202A in exon 10 of TYMP gene
Celebi et al, 2006	mutation in the TYMP gene (not further specified)
Çoban et al, 2013: 3 patients	N/A
Demaria et al, 2016	2 pathogenic TYMP mutations: c.687T>A and c.1160-1G>A
Durrani et al, 2019	homozygous mutation c.798_801dupCGCG in exon 7 of TYMP
Erdogan et al, 2019	novel homozygous c.765+1G>C intronic TYMP mutation
Feddersen et al, 2009	homozygous mutation c.605G>A,p.Arg202Lys in the ECGF1 gene
Gamez & Minoves, 2006	dup 18 pb/dup 18 bp in TYMP gene
	L371P/L371P in TYMP gene
	R44Q/R44Q in TYMP gene
	R44Q/R44Q in TYMP gene
Gramegna et al, 2018	homozygous c.1249 dupC TYMP mutation

	homozygous c.457G>A TYMP mutation
	homozygous c.1160–2A>G and c.1382_1383insC TYMP mutation
	homozygous c.215–1G>A and c.328C>T TYMP mutation
	homozygous c.1160–1G>A TYMP mutation
	homozygous c.522T>A TYMP mutation
	homozygous c.1160–1G>A TYMP mutation
Khan et al, 2022	N/A
Kripps et al, 2020	homozygous c.516+2T>A TYMP mutation
	compound heterozygous TYMP mutation: c.866A>C and c.653T>G
	homozygous c.214+1G>T TYMP mutation
Kučerová et al, 2018	homozygous c.647C>T in exon 6 of TYMP gene
Li et al, 2011	N/A
Libernini et al, 2012: 2 patients	2 heterozygous TYMP mutations: c.215–13_215delinsGCGTGA; c.1159 + 2T > A
Martín et al, 2004	compound heterozygous TYMP mutations: c.228G>A substitution, c.847C>G transversion, c.1311G>A transition and A465T polymorphism (c.1393G>A)
Massa et al, 2009	compound heterozygous TYMP mutation c.1160-1G>A and c.1135G>A
Millar et al, 2004: 5 patients	All TYMP mutations: 2/5: homozygous G1419A mutation, 2/5: homozygous T2294A mutation, 1/5: compound heterozygous mutations (A3371C and G3867C)

Nagata et al, 2017	N/A
Nalini et al, 2011	homozygous TYMP c.893 G>A mutation
Oztas et al, 2010	homozygous T5162C TYMP mutation (L371P)
Patel et al, 2019: 2 sisters	2 heterozygous TYMP variants: c.401C > A and c.845G > A
Peker et al, 2005	N/A
Petcharunpaisan et al, 2010	N/A
Rousset et al, 2008	compound heterozygote: exon2 C130T and exon9 G1283A
Said et al, 2005	Compound heterozygous substitutions of TYMP [Glu286Lys/Glu289Ala]
	Compound heterozygous substitutions of TYMP [Asn156Gly/Leu177Pro]
	Compound heterozygous substitutions of TYMP [Gly387Asn/Glu289Ala]
	Heterozygous Phe236Ser substitution of TYMP
Scarpelli et al, 2012	2 TYMP mutations: heterozygote c.199C>T and heterozygote c.866A>C
Scarpelli et al, 2013	Homozygous c.1160-1 G>A TYMP mutation
	Homozygous c.1160-1 G>A TYMP mutation
	Heterozygous c.866A>C; c.1231_1243del TYMP mutation
	Homozygous c.1160-1 G>A TYMP mutation
Schüpbach et al, 2007: 3 patients	All patients: homozygous g.2182C>A TYMP mutation (in exon 3)

Shaibani et al, 2009	c.329G>A and c.362G>A mutations of RRM2B
Suh et al, 2013	compound heterozygous TYMP mutations c.451A>C and c.809T>C
Tawk et al, 2020	heterozygous TYMP variants
Wang et al, 2016	homozygous TYMP mutation: duplication c.11930<br>1216dupGGGCGCTGCCGCTGGCGCTGGTGC
Yavuz et al, 2007	homozygous splice-site mutation IVS9-1G>A of TYMP gene
Zimmer et al, 2009	compound heterozygous TYMP point mutations c.261G > T & c.340G > A

Abbreviations. TYMP: thymidine phosphorylase gene
